# EPIDEMIOLOGICAL ANALYSIS OF FEMUR FRACTURES IN THE PEDIATRIC POPULATION IN A REFERENCE SERVICE IN THE STATE OF SERGIPE

**DOI:** 10.1590/1413-785220263402e293255

**Published:** 2026-05-11

**Authors:** Gustavo Henrique Cavalcanti Pereira Paixão, Thassyo Oliveira Sales, Gabriel Borges Paixão, Marcela Tavares Machado, Henrique de Sousa Monteiro, Mário Augusto Ferreira Cruz

**Affiliations:** 1 Universidade Tiradentes, Aracaju, Sergipe, SE, Brazil.; 2 Hospital Universitario, Lagarto, Sergipe, SE, Brazil.

**Keywords:** Epidemiological Profile, Femoral Fractures, Intramedullary Fracture Fixation, Perfil Epidemiológico, Fraturas do Fêmur, Fixação Intramedular de Fraturas

## Abstract

**Objective:**

To evaluate the clinical-epidemiological profile of skeletally immature patients treated at a referral service in Sergipe, from January to December 2023.

**Methods:**

This is an observational, retrospective study, by analysis of electronic medical records. The data were analyzed descriptively and for inferential analysis, the ANOVA and Student's t tests were used. Multivariate analysis was performed through logistic linear regression adjustment. A significance level of 5% was adopted, using R software, version 4.0.3.

**Results:**

Of the 49 femur fractures evaluated, 35 (71.4%) occurred in boys, 29 (59.2%) on the left side, with a mean age of 5.4 years. Three fractures were proximal (6.1%), 37 diaphyseal (75.5%) and 9 distal (18.4%). The most frequent fracture pattern was spiral (46.9%). It was found that the fracture mechanism and treatment presented significant differences in relation to age. Younger children were mainly affected by falls from their own height, while older children suffered direct trauma. In terms of treatment, increasing age behaves as a risk factor for surgical therapy.

**Conclusions:**

The data analyzed are equivalent to other studies performed and described in the world literature. **Level of Evidence III; Comparative Retrospective Study**.

## RESUMO

**Objetivo:**

Avaliar o perfil clínico-epidemiológico dos pacientes esqueleticamente imaturos atendidos em um serviço de referência em Sergipe, de janeiro a dezembro de 2023.

**Métodos:**

Trata-se de um trabalho observacional, retrospectivo, por análise de prontuários eletrônicos. Os dados foram analisados de forma descritiva e para a análise inferencial foram utilizados os testes ANOVA e t student. Foi realizada análise multivariada através do ajuste de regressão linear logístuca. Foi adotado um nível de significância de 5%, utilizando o software R, versão 4.0.3.

**Resultados:**

Das 49 fraturas de fêmur avaliadas, 35 (71,4%) ocorreram em meninos, 29 (59,2%) no lado esquerdo, com média de idade de 5,4 anos. Três fraturas foram proximais (6,1%), 37 diafisárias (75,5%) e 9 distais (18,4%). O padrão de fratura mais frequente foi em espiral (46,9%). Verificou-se que o mecanismo da fratura e o tratamento apresentaram diferenças significativas em relação a idade. As crianças mais novas foram principalmente acometidas por quedas da própria altura, enquanto as crianças mais velhas sofreram traumas diretos. No quesito tratamento, o aumento da idade se comporta como um fator de risco para terapêutica cirúrgica.

**Conclusões:**

Os dados analisados são equivalentes a outros estudos realizados e descritos na literatura mundial. **Nível de Evidência III; Estudo Retrospectivo Comparativo**.

## INTRODUCTION

The distribution of femoral fractures in children shows a bimodal pattern, related to anatomical characteristics and the stage of bone development, with fractures in early childhood generally resulting from low-impact trauma, while in adolescence, higher energy trauma is required.^1^ These fractures are classified according to their location: proximal, mid-diaphyseal, and distal. It is observed that, unlike adults, children have a preference for fractures in the middle third of the bone, or diaphyseal, accounting for the majority of these fractures (64%).^2-5^

These fractures, located in the diaphysis of the femur, represent between 1.5% to 2% of all fractures during the growth period, a relatively low incidence compared to more common fractures, such as those of the clavicle and wrist.^6^ However, they remain the main traumatic injury requiring pediatric hospitalization.^7-10^ The frequency of these fractures varies according to the studied region and seasonality, with a tendency to increase during the summer and late winter.^11^ Although there are few global epidemiological studies, a Swedish study indicated a rate of 11.3 cases per 100,000 individuals.^2^

The diagnosis of femoral fracture is straightforward, involving detailed physical examination and anteroposterior (AP) and lateral (P) radiographs. However, the possibility of associated injuries should be considered, especially in cases of high-energy trauma.^11^ The therapeutic approach varies according to age and fracture characteristics, with flexible intramedullary rods being widely used in children between 5 and 11 years old.^12,13^ However, in some cases, such as when specific resources are unavailable in the Unified Health System (SUS), alternative treatment methods should be considered.^14,15^

Given the limited number of comprehensive epidemiological studies, this study aims to evaluate the clinical and epidemiological profile of femoral fractures in skeletally immature patients in a reference service in the state of Sergipe.

## METHODS

This is a retrospective observational study conducted in a single reference institution for pediatric care in the state of Sergipe, based on the analysis of data extracted from electronic medical records. Patients under 13 years old who were treated in the service and diagnosed with femoral fracture were included in the study. Patients with pathological fractures or missing data were excluded from the study.

Data collection was performed by researchers using the computers of the Dr. José Machado de Souza Children's Hospital. Initially, electronic medical records of all patients treated by orthopedics from January to December 2023 were selected. Subsequently, each record was analyzed individually to identify cases of femoral fracture, and the radiographs of these selected patients were evaluated by the institution's digital radiography system. Finally, all previously selected records that were not excluded were stored in Excel spreadsheets (Microsoft®).

The variables considered in this study were: patient age, sex, race, municipality of residence, fracture laterality, trauma mechanism, fracture classification, fracture displacement pattern, associated fractures, type of treatment (surgical or conservative), specific treatment method, and early complications.

Initially, a descriptive analysis was performed. Categorical variables were described by absolute and relative frequencies, while quantitative variables were described by mean, standard deviation, minimum and maximum values. In the inferential analysis, the adherence to the normal distribution of quantitative variables was evaluated using the Shapiro-Wilk test.^16^ As normality of distribution was observed, ANOVA and the Student's t-test were used. The multivariate analysis was performed using Logistic Regression,^17,18^ with a log link function. The area under the ROC curve (AUC)^19,20^ was used to measure the model calibration, and the Hosmer-Lemeshow test^21^ was used to assess the model calibration. The results were expressed in terms of Odds Ratio (OR), calculated by the exponential of the estimated parameters. All conclusions from the hypothesis tests were based on the interpretation of the p-value, adopting a significance level of 5%. Whenever the p-value was less than 0.05, it was considered that there was a significant association between the analyzed variables. The software used for statistical analysis was R, version 4.0.3 (The R Core Team, 2024).

This study was approved by the Research Ethics Committee of the University of Tiradentes, under CAAE 76134323.8.0000.5371, according to opinion 7.041.777 and began after approval.

## RESULTS

**Figure 1:**
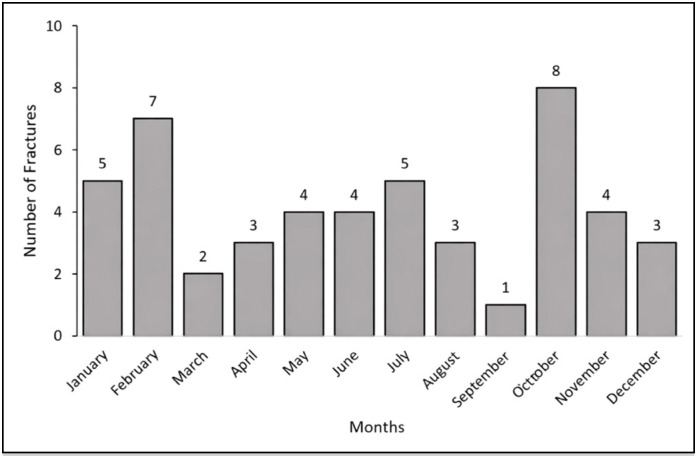
Monthly distribution of fractures.

The study population consisted of 49 children, with a mean age of 5.4 years. There was a predominance of the male sex (71.4%), of mixed ethnicity (81.6%), and from rural areas (63.3%). Figure 1 shows the monthly distribution of fractures.

The characteristics of the fractures (laterality, mechanism of injury, location and pattern of the fracture, and association with other injuries) are in Table 1. In addition to these data, no fracture from the present study was exposed.

**Table 1:** Characteristics and treatments of fractures.

Variable/Category	Frequency	Percentage (%)
**Side**		
Right	20	40.8
Center	29	59.2
**Mechanism**		
Motor vehicle collision	6	14.3
Fall from own height	18	42.9
Fall	14	33.3
Direct trauma	4	9.5
**Fracture location**		
Diaphysis	37	75.5
Distal	9	18.4
Proximal	3	6.1
**Fracture pattern**		
Articular	1	2.0
Wedge	3	6.1
Spiral	23	46.9
Oblique	6	12.2
Physeal (Salter Harris 2)	1	2.0
Torus	2	4.1
Transverse	13	26.5
**Other fractures**		
No	47	95.9
Distal radius	1	2.0
Proximal humerus	1	2.0
**Other injuries**		
No	48	98.0
Yes	1	2.0

Most patients underwent conservative treatment using plaster, and among the surgical techniques, fracture fixation with an external fixator was the most commonly used (Table 2).

**Table 2:** Characteristics of femur fracture treatment.

Variable/Category	Frequency	Percentage (%)
**Treatment**		
Surgical	23	46.9
Conservative	26	53.1
**Treatment method**		
External Fixator	9	18.4
Fio de Kirschner	1	2.0
Kirschner wire and external fixator	4	8.2
Plate	7	14.3
Screws	2	4.1
Plaster	25	51.0
Pavlik Harness	1	2.0

**Figure 2:**
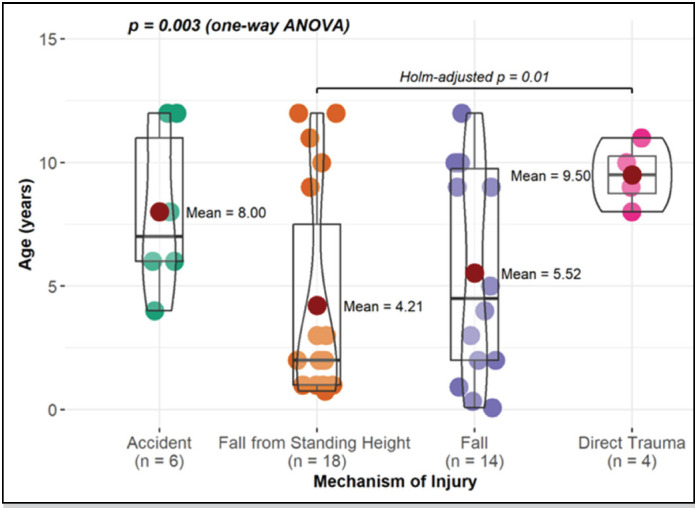
Comparison of age concerning the mechanism of injury.

**Figure 3:**
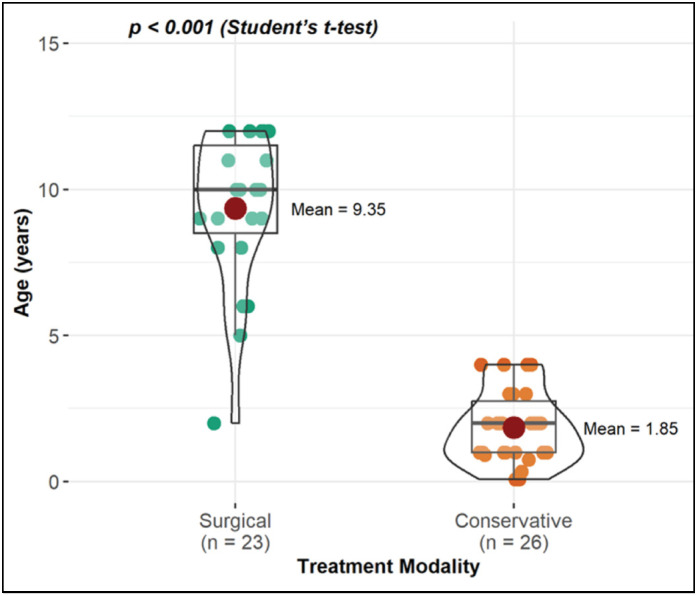
Comparison of age concerning fracture treatment.

When comparing age with sex and fracture location, no statistically significant difference was observed. However, when comparing age with the mechanism of injury and treatment performed, a statistical difference was found (Figures 2 and 3). Regarding the mechanism, when performing pairwise comparisons, it was found that there is a significant difference between the ages of patients with falls from their own height (FOH) and those with direct trauma. Thus, the average age of patients with the FOH fracture mechanism is statistically lower compared to those with direct trauma (Figure 2). With respect to the type of treatment, the average age of patients who underwent surgical treatment is higher than those with conservative treatment (Figure 3).

The result of the multivariate analysis through logistic regression is presented in Table 3. Among the variables analyzed in the model, it was found that only age is a risk factor for surgical treatment (p < 0.05). In other words, as the child's age increases, the chances of choosing surgical treatment are greater.

**Table 3:** Factors associated with surgical treatment.

Variables	Estimate	OR	CI 95%	P-value
Intercept	-6.58	0.00	(0.00; 0.08)	0.027
Gender (Male)	0.60	1.83	(0.03; 284.72)	0.783
Age	1.42	4.14	(2.04; 18.60)	0.006
Fracture Location (Distal)	-3.55	0.03	(0.00; 32.21)	0.496
Fracture Location (Proximal)	-2.27	0.10	(0.00; 46.67)	0.671

95% CI - 95% Confidence Interval; OR - Odds Ratio. The ROC curve (AUC) was 0.99. The Hosmer-Lemeshow test was 0.96.

## DISCUSSION

Our study identified a trend of predominance of fractures in the male sex, corroborating the findings of previous studies.^2,4^ Although our numbers are similar to those reported by Engström et al.^4^ the percentage of fractures in the male sex was slightly higher in our analysis. This difference may be attributed to the fact that we included only fractures resulting from trauma, excluding pathological, stress, and spontaneous fractures.

The bimodal age distribution observed in males is consistent with previous findings.^2,4,22^ The first peak occurs between 1 and 2 years of age, when children begin to walk, while the second peak is observed during higher energy activities, between 9 and 12 years. This second peak was not observed in females, which may be explained by social issues, such as lower exposure of girls to high-impact activities.

Regarding the location of fractures, there was a predominance of diaphyseal fractures, with results similar to those found in previous studies.^2-5^ Falls were identified as the main mechanism of trauma in our study, accounting for 76.2% of cases. This data is in line with previous studies,^2,4,6^ which also reported the predominance of this mechanism in younger ages. In older ages, however, traffic accidents are more common, especially in adolescents. This difference can be explained by the lower average age of the patients in our study compared to previous studies.

No specific seasonal pattern was observed regarding the incidence of fractures, although there was a predominance in the months of February and October. In contrast, Loder et al.^3^ reported a predominance in summer, while Von Heideken et al.^2^ observed a bimodal peak in winter/spring and summer. The absence of seasonal variation in our study can be attributed to the geographical location, where climatic differences and patterns of human behavior between the seasons are not as significant as in previous studies.

Regarding the type of treatment, the average age of patients undergoing conservative treatment was lower than that of those undergoing surgical treatment, which is in agreement with other studies.^4^ Our results suggest that a cutoff point of 5 years may be established, since 95.65% of fractures in children aged 5 years or older were treated surgically, while 100% of conservative treatments occurred in children below that age. This age is well established in the literature, especially for fractures located in the diaphyseal region, where, traditionally, until the age of five, the choice is for early reduction and cast immobilization, since what favors this type of treatment is the great potential for consolidation and remodeling, better tolerance to immobilization, and greater ease with general care, and after this age, fixation methods become more commonly used.^23, 24^

## CONCLUSION

The study covered demographic, epidemiological, and treatment aspects of femur fractures in children, contributing to guiding clinical practice and improving care for these patients. Moreover, the analyzed data are equivalent to other studies conducted and described in the global literature. Thus, we conclude that studies like this provide important information for health promotion policies and accident prevention for children.

## Data Availability

The contents underlying the research are available in the manuscript.
